# Light-Induced Increase of the Local Molecular Coverage on a Surface

**DOI:** 10.1021/acs.jpcc.4c00559

**Published:** 2024-04-01

**Authors:** Christophe Nacci, Donato Civita, Monika Schied, Elena Magnano, Silvia Nappini, Igor Píš, Leonhard Grill

**Affiliations:** † Department of Physical Chemistry, University of Graz, Heinrichstraße 28, 8010 Graz, Austria; ‡ CNR—Istituto Officina dei Materiali (IOM), Basovizza, 34149 Trieste, Italy; § Department of Physics, University of Johannesburg, P.O. Box 524, Auckland Park, Johannesburg 2006, South Africa

## Abstract

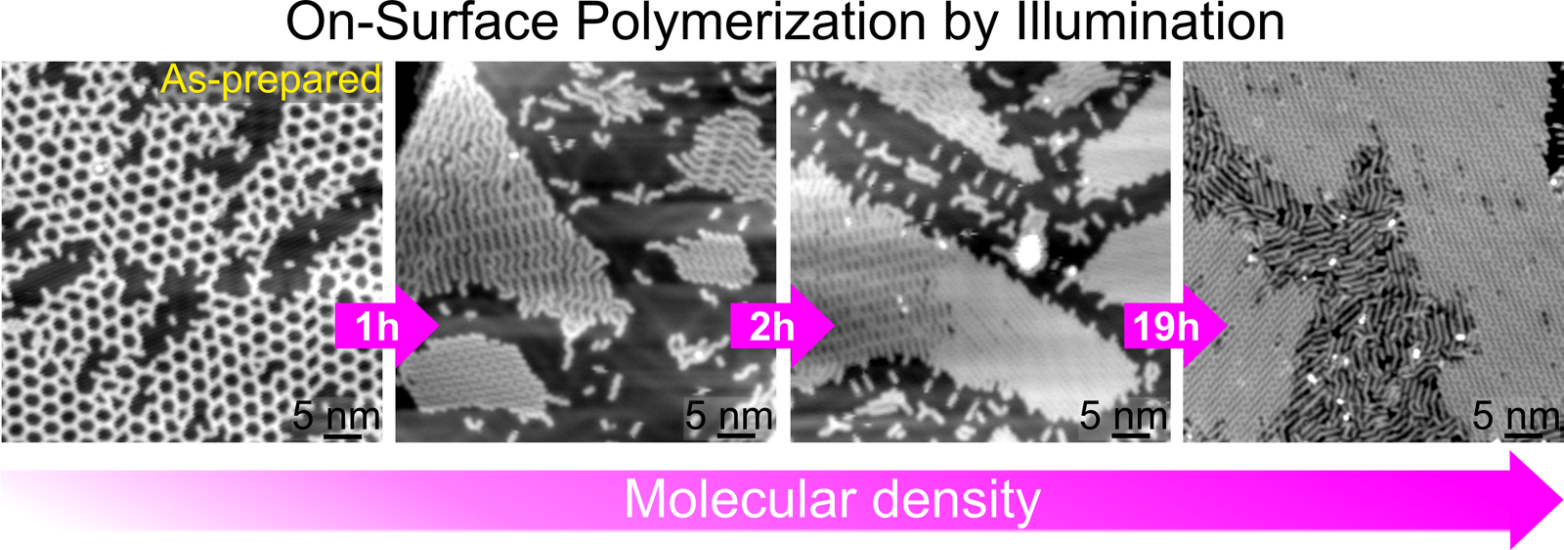

Light is a versatile tool to remotely activate molecules adsorbed on a surface, for example, to trigger their polymerization. Here, we explore the spatial distribution of light-induced chemical reactions on a Au(111) surface. Specifically, the covalent on-surface polymerization of an anthracene derivative in the submonolayer coverage range is studied. Using scanning tunneling microscopy and X-ray photoemission spectroscopy, we observe a substantial increase of the local molecular coverage with the sample illumination time at the center of the laser spot. We find that the interplay between thermally induced diffusion and the reduced mobility of reaction products steers the accumulation of material. Moreover, the debromination of the adsorbed species never progresses to completion within the experiment time, despite a long irradiation of many hours.

## Introduction

On-surface synthesis is a bottom-up scheme to grow stable covalent molecular architectures on solid surfaces, of interest in fundamental research^1−3^ as well as for applications^4,5^ that otherwise might not be accessible in solution chemistry.^1,6−8^ A crucial step when growing covalent structures is the activation of the adsorbed precursors, i.e., the formation of active sites within the molecules where the covalent linkage to other species will take place.^9^ Moreover, activated adsorbed species must diffuse efficiently across the surface to collide with each other, promoting the formation of new covalent bonds.

The on-surface synthesis of covalent structures has been achieved by various chemical reactions, as for instance Glaser coupling,^10^ Bergmann cyclization,^11^ imine coupling,^12^ dehydration of boronic acid,^13^ Diels–Alder reactions^14^ or Ullmann coupling of halogenated aromatics. The latter is one of the most preferred reactions^15−18^ with C–X (X = I, Br, ...) bonds at specific sites of the precursor molecule. The C–X bonds are typically the weakest within the molecular precursor,^19^ their position and spatial distribution therefore define where new C–C covalent bonds may be formed and consequently the geometry of the final molecular architecture.^9^

Coupling reactions have been initiated by different external stimuli: temperature,^2,20−22^ light,^23−27^ the tip of a scanning probe microscope^28^ (via voltage pulses), and electron beam irradiation.^29^ Although the dehalogenation of adsorbed molecular precursors has so far been mostly triggered by heat, light represents an attractive alternative tool because it is versatile and useable in different environments.^6^ The amount of energy to initiate the activation reaction can be carefully dosed by an accurate control of the photon frequency, photon density, and illumination duration. Accordingly, the different processes such as the activation of the adsorbed species and their diffusion across the surface can be efficiently decoupled from each other, in contrast to a thermal approach.^6^

Light-induced bond dissociation^30−32^ and formation^33,34^ have already been reported. The combined use of light and temperature was used to promote the growth of long poly *p*-phenylene polymers by mild heating.^35^ Long-range ordered two-dimensional (2D) covalent porous networks were obtained by [4 + 4] photocycloaddition of fluorinated anthracene triptycene on an alkene-passivated HOPG surface.^36^ Controlled long-range functionalization of a graphene layer was achieved by spatially selective photocycloaddition reactions in ultrahigh-vacuum (UHV).^37^ UV irradiation of self-assembled monolayers (MLs) of PCDA (C_25_H_42_O_2_) on graphene in UHV promoted the formation of one-dimensional conjugated backbone structures.^38^ We recently reported a comparative characterization of the light- and heat-driven polymerization of an anthracene derivative on Au(111)^39^ by scanning tunnelling microscopy (STM). The polymer length increases when on-surface polymerization is initiated by heating the sample at high temperatures. On the other hand, light-driven polymerization results mainly in the formation of rather short close-packed polymers whose lengths remain approximately unchanged with the illumination time.^39^ In this regard, the sample temperature plays a prominent role. All sample irradiations were conducted at room temperature (RT), i.e., in comparable conditions of molecular diffusion. At 77 K the polymerization process is suppressed as the debrominated species do not diffuse anymore while intact 2,6 dibromo-anthracene (DBA) molecules can still diffuse and assemble into compact structures.^39^

So far, most studies have focused on a structural characterization of the final products of the photochemical reactions by scanning probe microscopy.^23,40^ An investigation of the interplay between activation and diffusion when going from intact molecules to the covalently linked products is still largely unexplored. Moreover, there is a lack of knowledge about how these processes evolve spatially across the surface, with respect to the incident laser spot position.

Here, we study the elementary processes governing light-induced debromination and polymerization of an anthracene derivative on Au(111) by using STM and high-resolution X-ray photoemission spectroscopy (XPS). Experiments were conducted as a function of illumination time. Position-dependent measurements were also carried out to probe how the photochemical reaction products are spatially distributed across the surface. A key finding of this work is the significant light-induced increase in the molecular coverage in the surface area hit by the highest laser intensity.

## Methods

### Sample Preparation

The Au(111) sample was cleaned by repeated argon ion sputtering (*E* = 1.3 keV, sputtering time = 30 min, sample current = 5 μA) and subsequent heating at 770 K for about 10 min. DBA molecules were first degassed at 393 K for 20 min and then deposited from a Knudsen cell (sublimation temperature of 388 K, deposition time of about 15 s) onto Au(111) held at RT.

### STM Measurements and Sample Irradiation

STM images were recorded with a CreaTec low-temperature scanning tunneling microscope in constant-current mode between 5 and 7.5 K with the bias voltage referring to the sample with respect to the grounded STM tip. Irradiation of the samples was always done at RT in a preparation chamber under UHV. The beam spot position was identified by a ceramic sample plate that was placed in place of the Au(111) sample. A uniform bright round circle with a diameter of 6 mm was identified. Samples were illuminated in UHV with a CW solid-state laser (CryLas) with a nominal wavelength of 266 nm (power of 6.10 mW). The laser light entered into the UHV preparation chamber via a MgF_2_ UHV viewport that ensures a transmission coefficient of >90% in the 200–6000 nm wavelength range. The laser-sample distance was about 36 cm, and the sample illuminations were carried out in normal incidence condition. The sample temperature remained stable at RT during the illumination process (temperature was measured with a thermocouple). However, a small temperature increase in the surface area that was hit by the laser cannot be excluded.

### XPS Measurements and Sample Irradiation

High-resolution XPS spectra were carried out at the BACH^41,42^ beamline at the Elettra synchrotron (Trieste, Italy) in an UHV system composed of a preparation chamber and an analysis chamber equipped with a hemispherical electron energy analyzer (Scienta R3000, VG Scienta).^43^ A photon energy of 380 eV and a total instrumental resolution of 210 meV were employed to excite the C 1s and Br 3d core levels. All spectra were acquired at RT at an emission angle of 60° from the surface normal, and the intensities were normalized to the incident X-ray flux. The C/Br concentration ratio of 14:2, determined from the C 1s and Br 3d peak areas measured after DBA (C_14_H_8_Br_2_) deposition, confirmed that intact molecules were deposited on the Au substrate. To obtain the atomic concentration ratio, the peak areas were normalized to relative sensitivity factors, estimated as the product of the analyzer transmission function,^43^ theoretical photoionization cross-section,^44^ and the inelastic mean free path.^45^ All binding energies are stated relative to the Fermi level and calibrated by measuring the Au 4f core level spectra on a Au plate in electrical contact with the sample. The C 1s spectra were deconvoluted into Voigt lineshapes,^39^ while the Br 3d spectra were fitted using Voigt doublet lineshapes.^39^ Further information about the effects due to exposure to the synchrotron beam is included in the Supporting Information. Samples were illuminated in UHV with a CW solid-state-laser (CryLas) with a nominal wavelength of 266 nm (power of 6.10 mW). The brightest part of the incident beam spot was identified at the sample position by using a YAG/Ce scintillator as fluorescent material. This spot is approximately circular and has a diameter of about 1.5 mm. XPS measurements were taken at the center of this spot area. The X-ray beam has a diameter of about 0.4 mm. The laser light enters into the UHV preparation chamber via a fused Silica UV grade viewport (LewVac), ensuring a transmission coefficient of about 85% at 266 nm. Samples were approximately 80 cm away from the laser source. Illuminations were carried out with an incident angle of 22.5° from the surface normal. The sample temperature remained stable at RT during the illumination process, but (similar to the STM experiments) small temperature increases cannot be excluded.

## Results and Discussion

**Figure 1:**
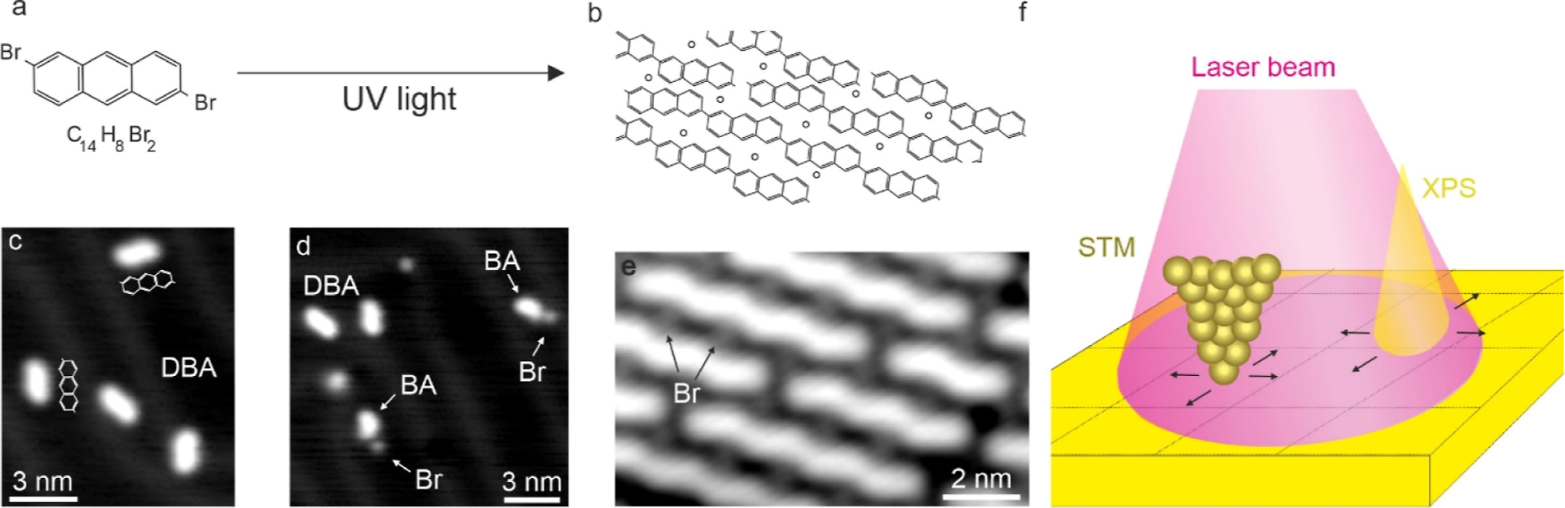
Polymerization process and experimental setup. (a) Chemical structure of the DBA molecule. (a,b) Light-induced polymerization process scheme: light-induced debromination and subsequent (b) formation of close-packed polymers separated by individual Br atoms. STM images of (c) intact DBA molecules, (d) partially debrominated DBA molecules, and (e) close-packed polymers separated by individual Br atoms [set points (c–e): 1 V and 20 pA]. (f) Position-dependent XPS and STM characterizations of the light-induced on-surface processes. The X-ray beam and the STM tip are displaced relative to the incident laser beam spot.

The molecular precursor DBA is carrying two bromine atoms in a trans configuration (Figure 1a) that promote the formation of linear covalent structures. Intact molecules appear in a shape that corresponds to their chemical structure^39^ (Figure 1c).

UV irradiation triggers the debromination of the adsorbed precursors^39^ (partially debrominated DBA molecules and Br atoms are shown in Figure 1d). Sample irradiation, which was in this work always conducted at RT, leads to the subsequent formation of close-packed islands of polymers typically separated by Br atoms (Figure 1b,e) and compact structures made of intact molecules (Figure S1). Light-induced on-surface synthesis has already been studied,^6,23,40^ including a statistical analysis of the polymer length distribution,^39^ but the spatial distribution of the chemical reactions with respect to the laser spot position is unclear. Here, we measure XPS spectra and STM images by changing the position of the X-ray beam or the STM tip with respect to the incident laser beam spot on the crystal surface to characterize the effect of progressive UV irradiation at different sample areas (Figure 1f).

**Figure 2:**
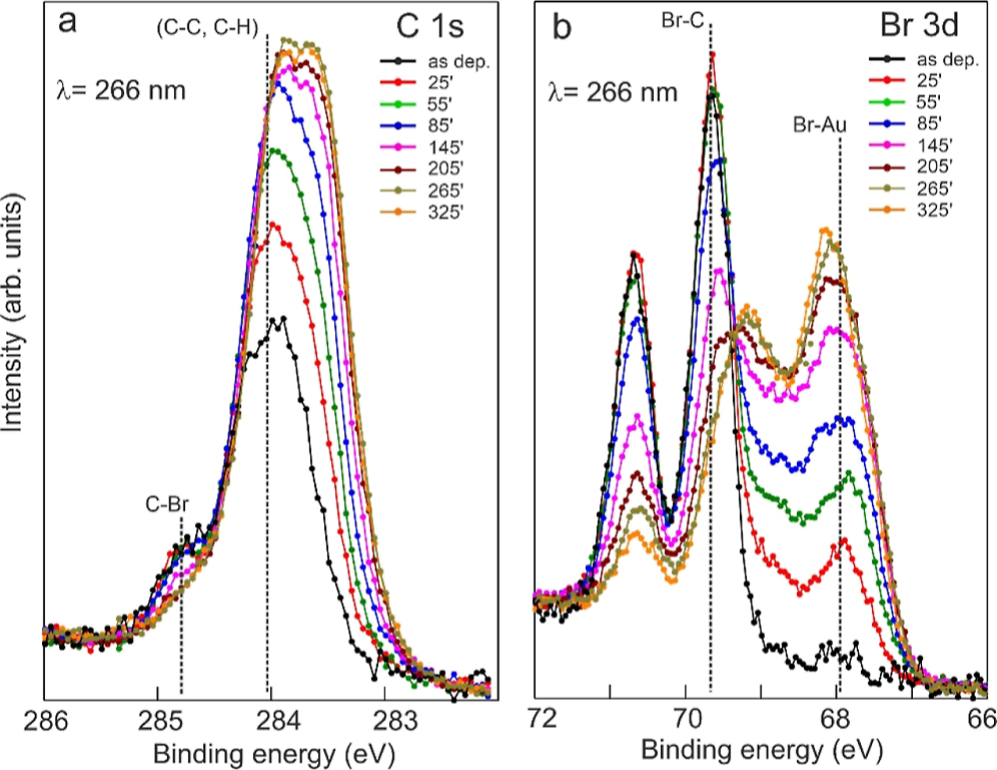
XPS characterization of the photoinduced polymerization. (a) C 1s and (b) Br 3d XPS spectra (*h*ν = 380 eV) of 0.5 ML DBA/Au(111) at RT as a function of the UV (266 nm) illumination time. Spectra were recorded at the center of the *I*_max_ laser spot. The UV illumination time (in minutes) is indicated and the spectra are color-coded accordingly.

Half a ML of DBA molecules was deposited onto Au(111) kept at RT. Light-induced debromination and reactions of the adsorbed species were first studied by probing the C 1s and Br 3d core level spectra (see Figure 2) as a function of the illumination time at the surface area where the laser beam shows its brightest spot, i.e., the highest intensity (henceforth *I*_max_ spot; see also Section S2 and Methods). Surprisingly, the C 1s intensity increases with the illumination time (Figure 2a) and also becomes broader (see Section S2 of the Supporting Information). The peak shoulder at high binding energy (BE), representing the C–Br component,^46,47^ is progressively depleted due to the ongoing light-induced debromination process. This is further corroborated by the significant change in the Br 3d core level. Upon irradiation two new spectroscopic features emerge at low BE^48,49^ at the expense of the covalent Br–C component (Figure 2b), indicating more chemisorbed bromine atoms on the Au surface. From a quantitative point of view, the C 1s intensity roughly doubles after about 3 h of irradiation and remains almost stable afterward (Figure S2a). The Br 3d intensity also increases within the first stages of the UV illumination and then unexpectedly decreases slowly afterward (see Figure S2a). The Br loss might be related to either Br atoms diffusing out of the irradiated area or photoinduced Br desorption (see Supporting Information). The increase in the C 1s signal intensity with illumination is ascribed to the progressive accumulation of DBA-based structures at the *I*_max_ spot, i.e., the increase in the local molecular density on the surface. A close inspection of the photoemission spectra shows that C 1s undergoes a downshift of about 0.30 eV with the progressive illumination (Figure 2a). A similar shift has been already observed for thermally driven polymerization and was ascribed to the work function increase of the surface, due to chemisorbed Br atoms.^46,47^ A stronger interaction with the substrate and partial orbital delocalization along the covalent anthracene chains might contribute to the line shapés broadening.^46,50^ The debromination process is furthermore never completed in the experimental time as a covalent Br–C component is always present, even after 325 min of UV irradiation (Figures 2b and S2b).

**Figure 3:**
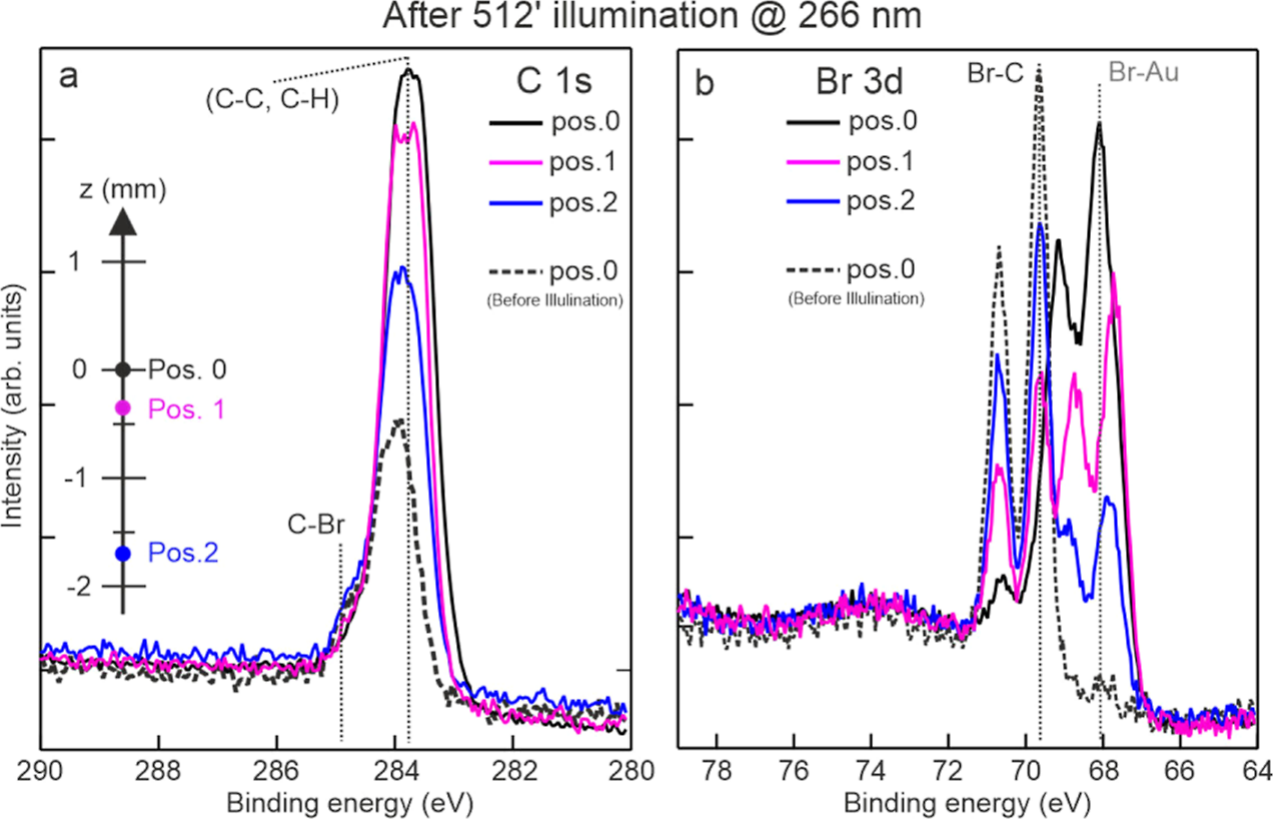
Position-dependent XPS measurements of light-induced on-surface polymerization. C 1s (a) and Br 3d (b) XPS spectra (*h*ν = 380 eV) were acquired at different positions after 512 min of illumination with UV (266 nm) laser light [position 0 is at the *I*_max_ spot center-this is approximately circular and has a diameter of about 1.5 mm (see Methods)]. The spectra are color-coded, and the respective positions compared to laser spot center are indicated in panel (a). The components of C 1s and Br 3d and their energy positions are indicated. To better highlight the light-induced changes, C 1s and Br 3d core levels are also shown at position 0 before the irradiation (dashed black curve).

In order to explore the origin of this molecular coverage increase, XPS measurements were also conducted at surface areas off the *I*_max_ spot after long irradiation of the surface (512 min of UV illumination, see Figure 3). The C 1s and Br 3d core level intensities decrease when probed away from the *I*_max_ spot (Figures 3 and S3), which confirms that the molecular coverage increase is higher at the *I*_max_ laser spot center. Furthermore, the debromination is also here more pronounced compared to that of the surrounding (Figure S3). The spatial variation of debromination and polymerization across the surface also results in a different position-dependent energy shift of the C 1s and Br 3d core levels (Figure 3) relative to the energy positions before the illumination (see Supporting Information and references therein). These experimental findings show that species activation is more efficient at the center of the *I*_max_ spot owing to the larger photon density as compared to the surrounding area. Accordingly, the debromination rate as well as the accumulation of material both strongly decrease with the distance from this spot area.

**Figure 4:**
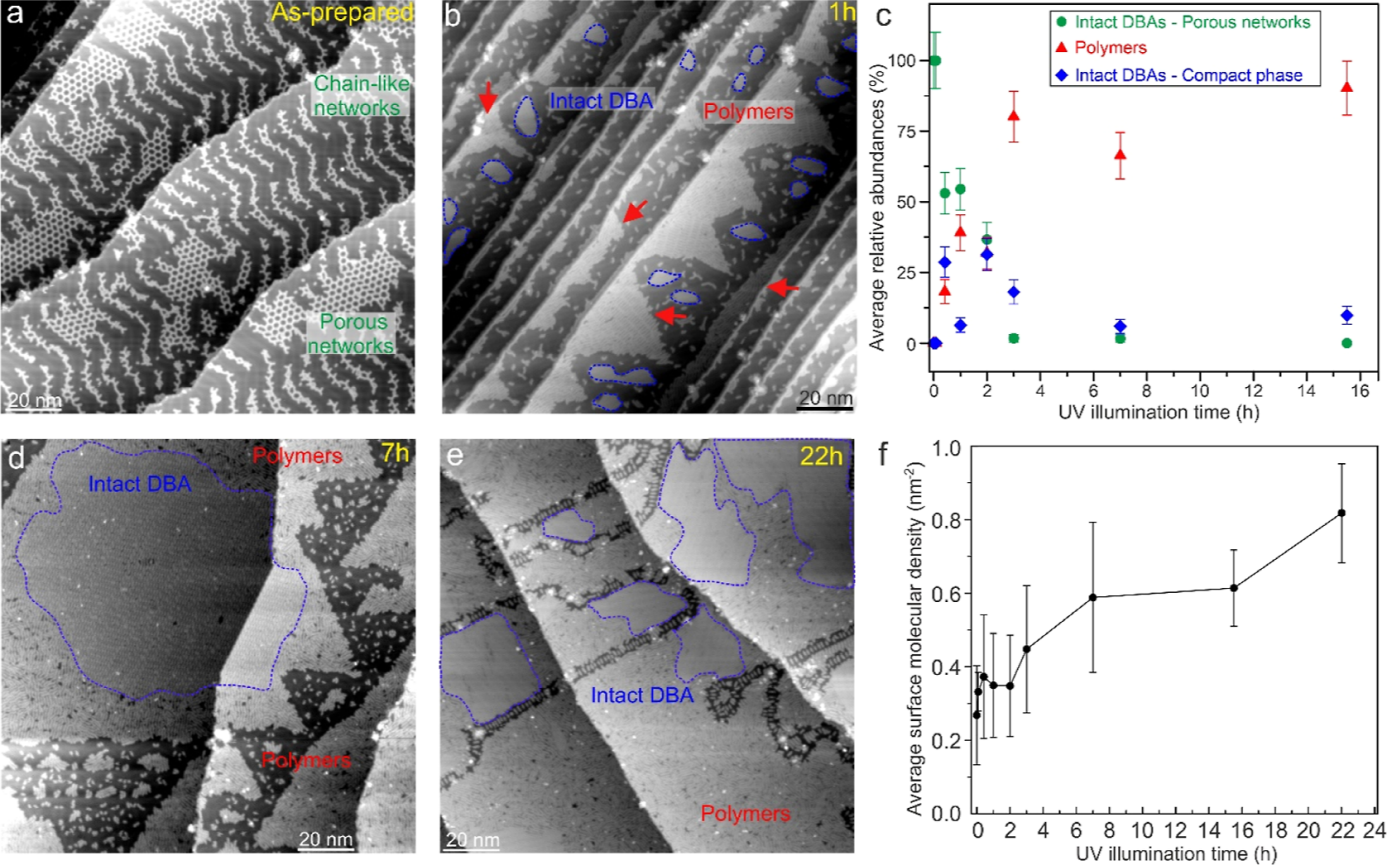
Illumination time-dependent STM measurements. Series of representative overview STM images, all 147.1 nm × 147.1 nm in size [(a) 1 V, 30 pA; (b) 1 V, 20 pA; (d) 1 V, 11 pA and (e) 1 V, 13 pA], taken at the center of the incident laser beam on the surface, i.e., point 0 in the sketch shown in Figure S4a: as-prepared (a), after 1 h (b), 7 h (d), 22 h (e). Extended compact structures made of intact DBA molecules are highlighted by dashed blue lines. (c) Average relative abundances (in %) of intact DBA porous networks (green circles), compact intact DBA clusters (blue squares), and polymers clusters (red triangles) as a function of the illumination time. (f) Average surface molecular density for different illumination times. Each experimental point in panels (c,f) is the average value estimated from 20 to 30 different STM images. The corresponding error bar amplitude is determined by the standard deviation of these values [relative abundances in (c) and molecular density in (f)].

In Figure 4a we report an STM overview of the sample as prepared. The adsorbed DBA molecules are mostly intact and arranged into either 2D porous network or chain-like structures following the Au herringbone reconstruction all across the crystal surface. To explore how these networks evolve with illumination, STM images were taken as a function of the photon dose (i.e., illumination time) and the surface area position with respect to the incident laser beam. In the former case the STM imaging was carried out at the center of the incident laser spot on the surface (Figure S4a). The formation of close-packed islands of intact molecules (Figure S1) or polymers (Figure 4b) is already observed after rather short (25 min) UV irradiation. The evolution of surface areas covered with intact DBA-based clusters and polymers arrangements (Figure 4c) was studied by a quantitative analysis of the STM images from the as-prepared surface (Figure 4a) to the extensively irradiated surface (22 h in Figure 4e; see also Figure S4).

Intact DBA species arranged in porous networks are turned into a compact phase structure within the first hours of illumination (Figure 4c). The compact phase of DBA molecules was also previously observed when annealing the sample at moderate temperatures.^39^ Furthermore, a steady increase of the polymer population takes place at the expense of the intact DBA molecules, owing to the ongoing debromination/polymerization process, until a plateau is reached (Figure 4c). The significant increase of the local molecular surface density with the illumination time is evident in a sequence of STM overview images of the surface, ranging from as-prepared to long illumination periods, as shown in Figure 4 (see Figure S4 and zoom-in STM images in Figures S5–S7 for more structural details). The average surface molecular coverage remains stable around 0.35 nm^–2^ within the first few hours of illumination (Figure 4f). Only afterward does it start to increase slowly until it is roughly doubled after 22 h of illumination. From a qualitative point of view, both STM and XPS experimental findings consistently reveal a significant photoinduced increase of the local molecular surface density at the center of the laser spot. Furthermore, the photoactivity on the surface is observed to significantly decay when moving off of this area due to the lower photon density. From a quantitative point of view, the accumulation of material at the *I*_max_ spot apparently takes place on a different time scale in STM and XPS experiments. We ascribe this discrepancy to the fact that these experiments were conducted in different experimental setups and geometries.

The technical difficulties in addressing exactly the same sample area (relative to the laser spot) hamper a straight quantitative comparison between STM imaging and XPS spectra. Position-dependent STM measurements after long (21 h) UV illumination reveal a variety of distinct DBA-based structures across the illuminated surface area (Figure S8). Before irradiation (Figure S8a) porous networks made of intact DBA molecules are found all over the surface. After irradiation, laterally extended islands of close-packed polymers and compact assemblies of intact DBA molecules are mainly found at surface areas close to the laser beam center (Figure S8d–f). Furthermore, at the rim of the illuminated surface area, intact molecules are arranged into a compact phase and found more often than porous networks (Figure S8b,c). These structural findings highlight a pronounced photoinduced coverage increase at the most irradiated surfaces areas (laser spot center). Away from these areas a reduction of the debromination/polymerization activity is observed. These findings qualitatively corroborate the position-dependent photoemission spectra shown in Figure 3.

Despite the rather long UV illuminations used in this work, debromination of the adsorbed DBA species is not brought to completion. Compact structures composed of intact DBA molecules are still found after a significant dosing time (up to 22 h) of UV illumination (Figures 4e and S7). The C 1s and Br 3d core levels taken at the *I*_max_ spot contain a clear residual covalent C–Br component (Figures 2 and S2) even after several hours of UV illumination. In this regard, the spectroscopic evolution of the covalent Br 3d component (Br–C) with the illumination time suggests an exponential decay of the population of intact DBA to a finite nonzero asymptotic value (Figure S9). This asymptotic value has been determined for DBA coverages of 0.5 and 1.0 ML: it is about 6% for 0.5 ML coverage and is significantly increased up to about 22% when the coverage is doubled to a full ML (Figure S9). This suggests that the DBA coverage affects the photoinduced debromination rate. We tentatively ascribe the reduced occurrence of the photoinduced debromination to steric constraints that might become more prominent at higher molecular coverages. The fact that the debromination is never completed might also be ascribed to an equilibrium between the debromination and rebromination processes.^51^ Additionally, kinetic effects come into play as monomers might be trapped in certain surface areas by polymer structures and can thus not contribute to further growth (Figures 4 and S4–S7).

The light-driven increase in the local molecular coverage is a common feature observed for different DBA coverages on the Au(111) surface. At low molecular coverage of about 0.1 ML, the as-prepared surface shows small molecular clusters composed of few DBA molecules (Figure S10a). After 12 h of continuous UV illumination, extended clusters (hundreds of nm large) are observed by STM at the surface areas corresponding to the laser beam spot center (Figure S10). The same trend is observed (with XPS) at higher molecular coverages. The light-driven increase of the C 1s and Br 3d core level intensities is also observed at 1.0 ML (Figure S11). After 11 h of UV illumination the C 1s intensity increases by a factor of 1.2 at the *I*_max_ spot (Figure S11c). This is less than that for the case of 0.5 ML DBA/Au(111) in comparable illumination conditions (Figure S2), owing to the high initial molecular coverage.

We assign the light-induced increase in the molecular coverage to two simultaneous processes: the diffusion of the adsorbed species and the ongoing light-induced debromination. All UV irradiations were conducted with the sample kept at RT. This means that DBA molecule keeps diffusing across the surface (Figure S12), also in illuminated areas, and the photoactivated molecules mainly react with each other, resulting in the formation of polymers. Laser-induced local heating might enhance the molecular mobility even further. Importantly, the diffusion barrier of molecules at surface typically increases with their molecular size,^1,24^ thus lowering polymer diffusion as compared to single monomers and reducing the number of collisions. Thus, the local accumulation of DBA-based species in the laser spot on the surface is ascribed to the continuous supply of monomers from “dark” surface areas into the laser spot where they get trapped by polymerization.

## Conclusions

In summary, this study gives insights into the light-driven Ullmann coupling of anthracene derivatives on Au(111). Molecules on the surface were irradiated and then investigated by combining microscopic and spectroscopic information. Structural and spectroscopic characterizations demonstrate a significant increase in the local molecular coverage with the illumination time at the surface areas exposed to the most intense light. STM imaging reveals large close-packed assemblies at the most irradiated surface areas. Porous molecular networks identified before the illumination are frequently observed at surface sites rather remote to the laser spot center that are still irradiated but with lower intensity. This is a novel and nonobvious effect caused by the interplay between effective light-induced debromination and thermally driven molecular diffusion. Moreover, the light-driven debromination is affected as the photoactivation rate of adsorbed DBA species decreases at higher coverages, which is assigned to steric constraints and possibly to the recombination between activated species and diffusing bromine atoms.

## Supporting Information Available

DBA/Au(111)—compact phase; C 1s and Br 3d core level peak areas vs illumination time −0.5 ML DBA/Au(111); position-dependent XPS of C 1s and Br 3d core levels; STM imaging after different UV illumination times; STM imaging after 3 h of UV illumination −0.5 ML DBA/Au(111); STM imaging after 7 h of UV illumination −0.5 ML DBA/Au(111); STM imaging after 22 h of UV illumination −0.5 ML DBA/Au(111); position-dependent STM imaging −0.5 ML DBA/Au(111); percentage of intact DBA molecules vs UV illumination time; position-dependent STM imaging −0.1 ML DBA/Au(111); C 1s and Br 3d core levels vs illumination time −1.0 ML DBA/Au(111); STM imaging of DBA/Au(111) at different temperatures; and effects due to exposure to the synchrotron beam (PDF)
