# Supplementary material for: Light-Induced Increase of the Local Molecular Coverage on a Surface
Source: J Phys Chem C Nanomater Interfaces. 2024 Apr 1;128(14):5919–26. doi: 10.1021/acs.jpcc.4c00559 (PMC11017312; doi:10.1021/acs.jpcc.4c00559)
Supplement: Supplementary file 1 — jp4c00559_si_001.pdf [file jp4c00559_si_001.pdf]

Light-Induced Increase of the Local Molecular Coverage on a Surface

Christophe Nacci*[,1], Donato Civita[1], Monika Schied[1,†], Elena Magnano[2,3],

Silvia Nappini[2], Igor Píš [2], and Leonhard Grill*[,1]

1) Department of Physical Chemistry, University of Graz, Heinrichstraße 28, 8010 Graz, Austria

2) CNR - Istituto Officina dei Materiali (IOM), 34149 Basovizza (TS), Italy

3) Department of Physics, University of Johannesburg, P.O. Box 524, Auckland Park, Johannesburg 2006, South Africa

†Elettra Sincrotrone Trieste S.C.p.A., Strada Statale 14 Km 163.5, Trieste, 34149 Italy

*Corresponding authors: christophe.nacci@uni-graz.at and leonhard.grill@uni-graz.at

Table of contents

1. DBA/Au(111) – Compact phase S2
2. C 1s and Br 3d core level peak areas vs illumination time - 0.5 ML DBA/Au(111) S3
3. Position-dependent XPS of C 1s and Br 3d core levels S5
4. STM imaging after different UV illumination times S6
5. STM imaging after 3 hours of UV illumination - 0.5 ML DBA/Au(111) S7
6. STM imaging after 7 hours of UV illumination - 0.5 ML DBA/Au(111) S8
7. STM imaging after 22 hours of UV illumination - 0.5 ML DBA/Au(111) S9
8. Position-dependent STM imaging – 0.5 ML DBA/Au(111) S10
9. Percentage of intact DBA molecules vs UV illumination time S11
10. Position-dependent STM imaging – 0.1ML DBA/Au(111) S13
11. C 1s and Br 3d core levels vs illumination time – 1.0 ML DBA/Au(111) S14
12. STM imaging of DBA/Au(111) at different temperatures S15
13. Effects due to exposure to the synchrotron beam S16
14. References S16

1. DBA/Au(111) – Compact phase

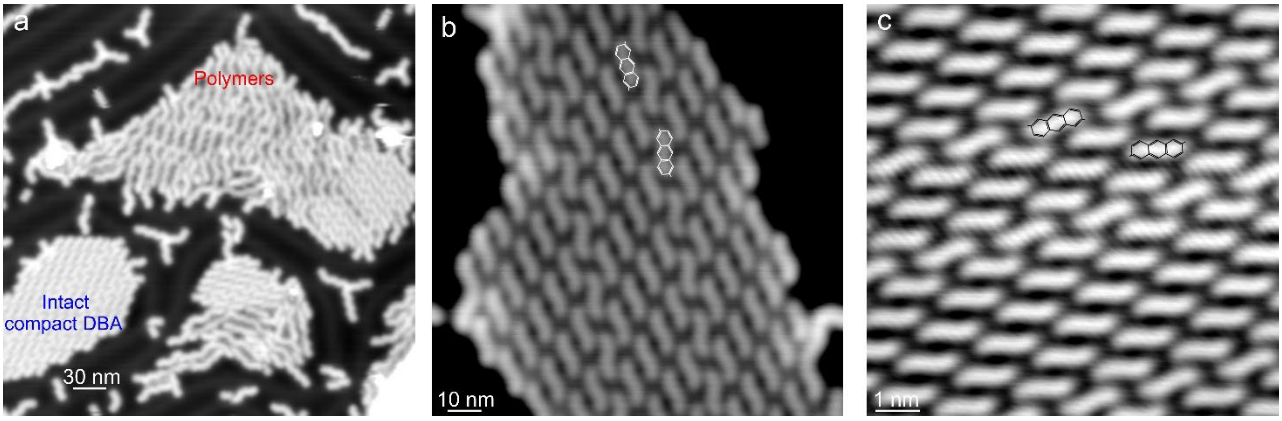

Figure S1. (a) Large STM overview of 0.5 ML DBA/Au(111) after 25 minutes of illumination at 266 nm. Images taken at the center of the incident laser spot. Islands comprised either of intact DBA molecules or polymers are observed. (b,c) Zoom-in of the DBA compact phase on Au(111). The molecule chemical structure is superimposed to highlight the two different molecular orientations. Setpoints: (a: 0.5 V, 60 pA; b: 0.1 V, 100 pA; c: 0.1 V, 400 pA).

Intact DBA molecules deposited onto Au(111) at room temperature (RT) results in the formation of extended porous networks and chain-like structures (Fig.4a). After short UV illumination (25 minutes) part of the porous networks is turned into a compact phase (a). (b,c) Different molecular domains of molecules can be observed within the compact phase. The imaging contrast change across the polymer clusters is more prominent in comparison to the compact molecules (a).

2. C 1s and Br 3d core level peak areas vs illumination time - 0.5 ML DBA/Au(111)

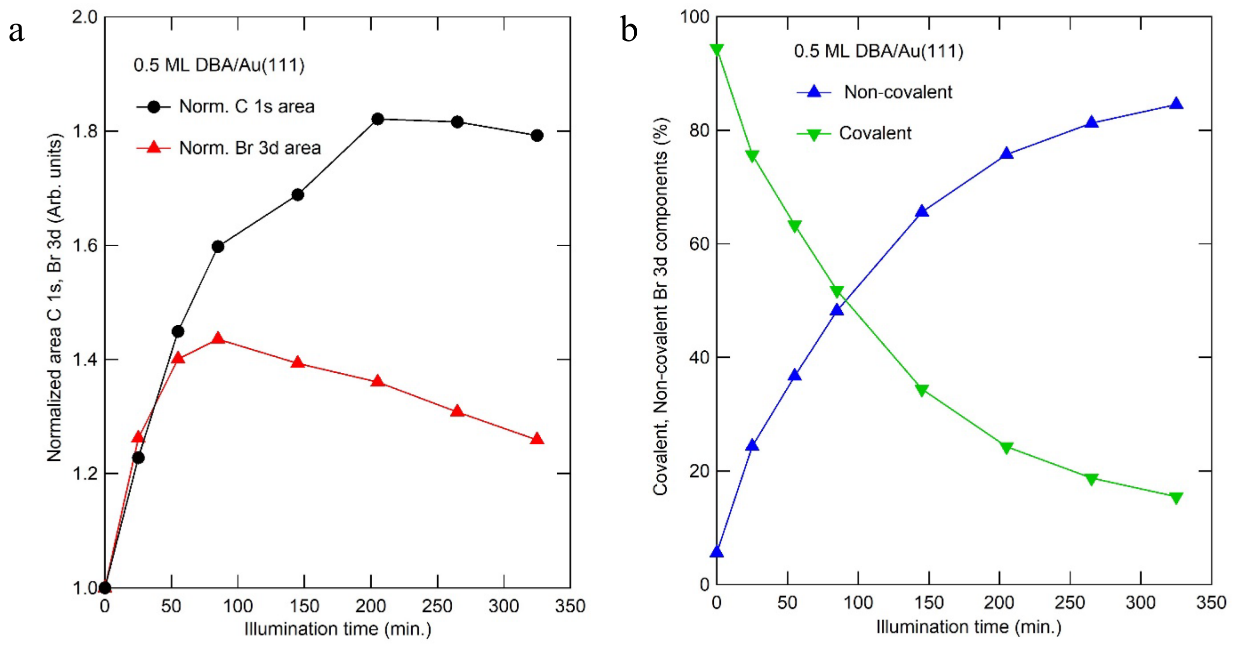

Figure S2. (a) Normalized peak area of C 1s and Br 3d core levels taken on 0.5 ML DBA/Au(111) as a function of the illumination time. Measurements taken at the I_{max} spot center at room temperature. The peak areas have been normalized to the corresponding area values measured at time zero, i.e., before the UV illumination. (b) The covalent (Br–C, green) and non-covalent (Br/Au(111), blue) Br 3d component normalized areas are shown as a function of the illumination time. Both component areas are normalized to the entire Br 3d core level area.

The brightest laser spot (I_{max} spot), which was identified by using a YAG:Ce scintillator (see Methods), has a diameter of about 1.5 mm. XPS measurements, *i.e.* C 1s and Br 3d core level studies, were done at the center of the I_{max} spot. All measurements were conducted at RT. The core level peak areas are normalized to the corresponding peak area before the illumination.

In Figure S2a, the normalized peak areas of both core levels are plotted against the illumination time. The areas of the C 1s peaks were deduced by fitting individual spectra. The C 1s spectra were deconvoluted as done previously[1], *i.e.*, using three Voigt lineshapes to take into account the different component contributions (C-Br: carbon atoms covalently bound to Br atoms, C-C and C-H: anthracene unit). The C 1s peak area roughly doubles after about 3 hours of UV illumination. Any further illumination left the C1 s peak area unchanged – see plateau in panel (a).

The Br 3d signal has a very different behavior. It first reaches a maximum after ~90 minutes of illumination (Fig.S2a), this corresponds to an increase by about a factor of 1.4 compared to the non-illuminated case. Afterwards, a slow decrease of the Br 3d peak is unexpectedly observed (about -12% in 4 hours). The Br intensity loss is also observed at higher molecular coverage (see S11). Br atoms diffusing out of the investigated area might account for the observed reduction of Br intensity. In this regard an equilibrium between species diffusing in and out of the investigated area seems to be a plausible scenario. Br atoms diffusing around might be photo-desorbed through the formation of Br_2. Such a process is already known to take place during the thermally-assisted on-surface synthesis with brominated species[2,3]. Other possible Br loss mechanisms might be due to the combination of

Br atoms with atomic hydrogen either present in the chamber or from dehydrogenated species[4]. We believe that these processes play a minor role here[1].

The Br 3d spectra were fitted using Voigt doublet line shapes as done previously[1]. Two major contributions are identified (Fig.S2b): a covalent component caused by Br atoms covalently bound to molecules (green) and a non-covalent one at low BE ascribed to chemisorbed Br atoms on Au(111) (blue). The latter is typically fitted with two components[1,5,6]: the first component is ascribed to chemisorbed Br atoms on Au(111) while the second component assignment is still debated[1,5,6]. The need for two fitting components might be related to Br atoms in different Au adsorption sites (for instance, *fcc* and *hcp*) as well as to Br_2 or Br clusters with different charge states[1,5,6]. However, STM imaging does not reveal any Br clusters and Br_2 should not even remain stable or stick on the Au surface at room temperature[7]. Among all possible scenarios, a chemical shift due to Br atoms sitting on different chemical environments seems to be the most plausible one.

The on-going debromination process causes the progressive depletion of the covalent component at high BE. After 325 minutes of UV illumination the debromination process is still not completed. Indeed, about 20% of the Br 3d signal still originates from the molecule-bound Br atoms (see green curve in Fig.S2b). For further discussions see S11-12.

Moreover, a progressive shift and tightening of the non-covalent Br 3d component is observed too (see Fig.2b). This is further highlighted by the position-dependent XPS measurements (Fig.3). The C 1s core level shape reveals also a broadening with the illumination time (Fig.2a): its FWHM is (875 ± 25) meV before illumination, increasing up to (888 ± 25) meV after 85 minutes and (997 ± 25) meV after 325 minutes of UV illumination. The broad C 1s component at about 284 eV (see black curve in Fig.2a, before the illumination) is related to the aromatic carbon atoms in the anthracene units[8,9]. The progressive accumulation and coexistence of clusters of polymers with different length and intact DBA units significantly enhances the contributions of the C–C and C–H components[1]. The C–C component is further boosted because of the formation of new covalent carbon bonds owing to the ongoing polymerization.

3. Position-dependent XPS of C 1s and Br 3d core levels

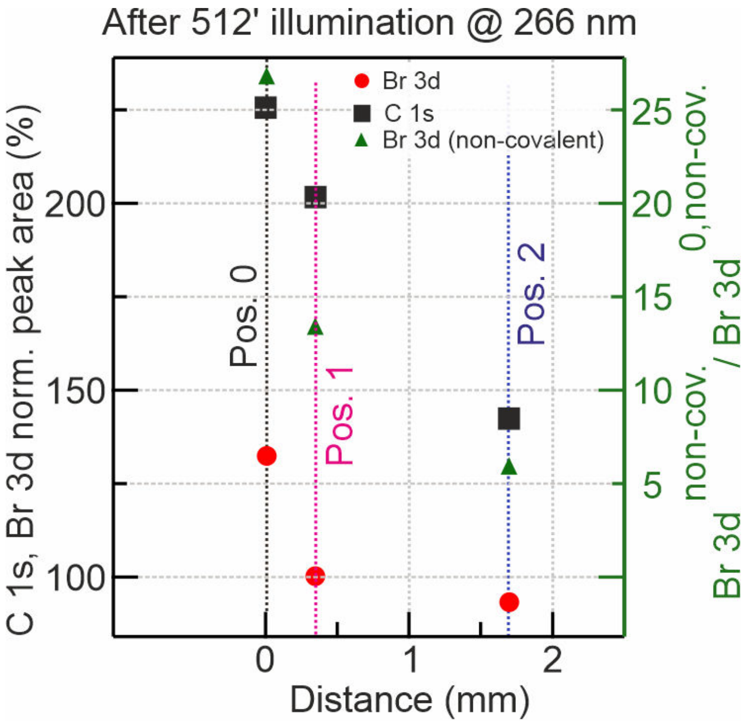

Figure S3. Normalized peak area of C 1s and Br 3d acquired at positions **0**,**1** and **2** as shown in Fig.3a in the main text. Position **0** is the center of the I_{max} spot (diameter of about 1.50 mm). The areas are normalized to the respective core level areas measured at position **0** before the illumination. The non-covalent Br 3d component (chemisorbed Br atoms on Au(111)) is also shown (see green triangles) and normalized to its value at position **0** before the illumination.

XPS measurements of C 1s and Br 3d core levels were carried out at few different surface areas (see positions **0, 1 and 2** in Fig.3) after 512 minutes of irradiation with UV (266 nm) laser light. All measurements were conducted at RT. The peak areas of C 1s and Br 3d core levels are normalized to the respective peak area at position **0** before the illumination.

4. STM imaging after different UV illumination times

DBA molecules deposited on Au(111) are found arranged in porous networks and linear structures that follow the herringbone reconstruction (Fig.S4a) when STM imaged at 7.5 K. After few hours of UV (266 nm) illumination extended close-packed structures comprised of intact DBA molecules and polymers are found (Fig.S4b-d). The lateral extension of these islands increases with the illumination time (7 hours (Fig.S4e), 22 hours (Fig.S4f)). All STM images in Fig.S4 were taken at surface areas corresponding to the laser spot center (see inset of panel (a), laser spot diameter of about 6 mm) and have the same size (147.1 nm × 147.1 nm). This further highlights the increase of the local molecular density with the illumination time - cf. panels (a) and (f).

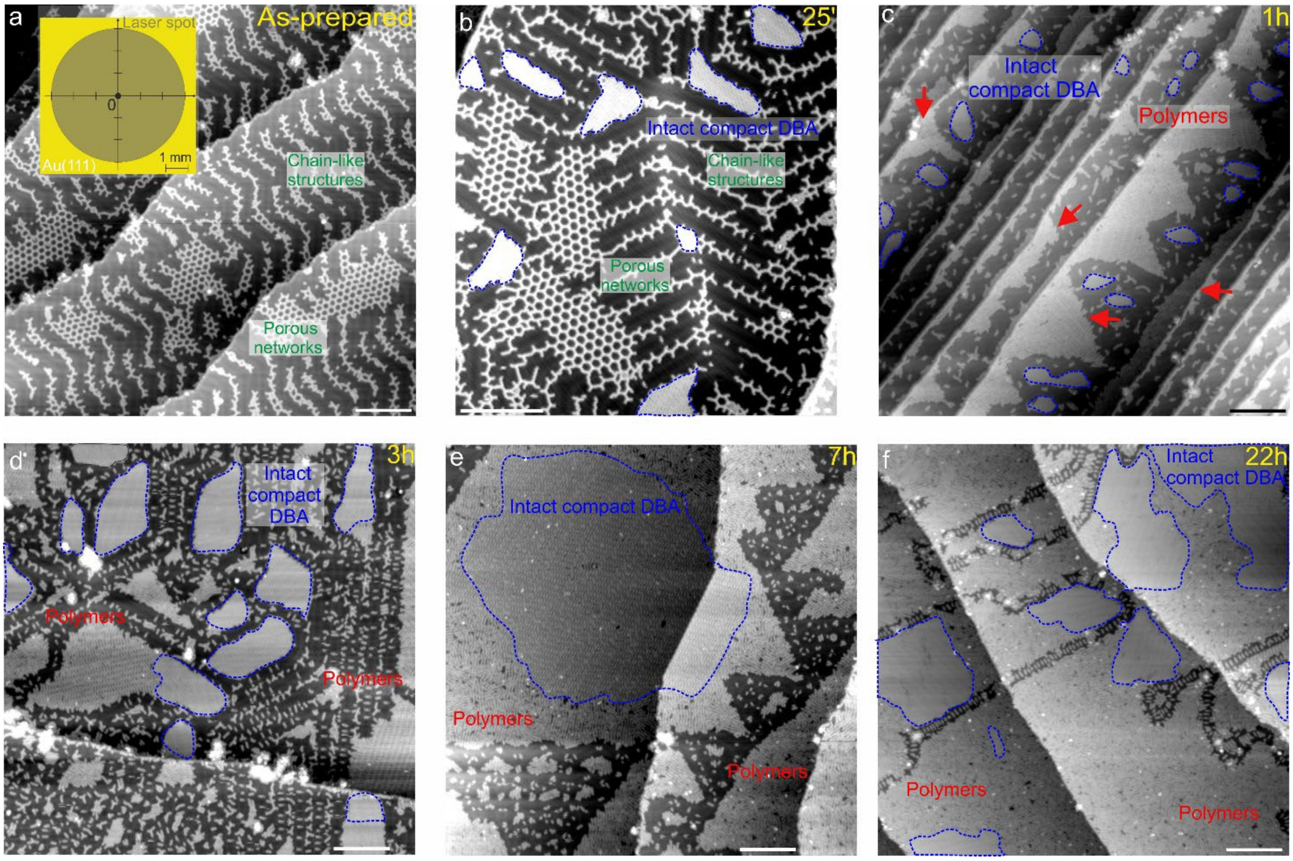

Figure S4. (a) Series of representative large STM overview of the 0.5 ML DBA/Au(111) taken at the center of the laser spot on the surface (point **0** in the inset of panel (a)) after different UV (266 nm) illumination times: 25 min (b), 1 h (c), 3 h (d), 7 h (e) and 22 h (f). All STM images have the same size, (147.1 × 147.1 nm^2). Setpoints: bias voltage= 1 V, tunnel current: a: 30 pA; b: 20 pA; c,f: 13 pA; d: 14 pA; e: 11 pA. Intact DBA arranged in porous networks (green) and compact structures (blue) and polymers (red) are indicated in all STM images. All irradiations were conducted at RT. STM operated at 7.5 K. The scale bar is 20 nm.

5. STM imaging after 3 hours of UV illumination - 0.5 ML DBA/Au(111)

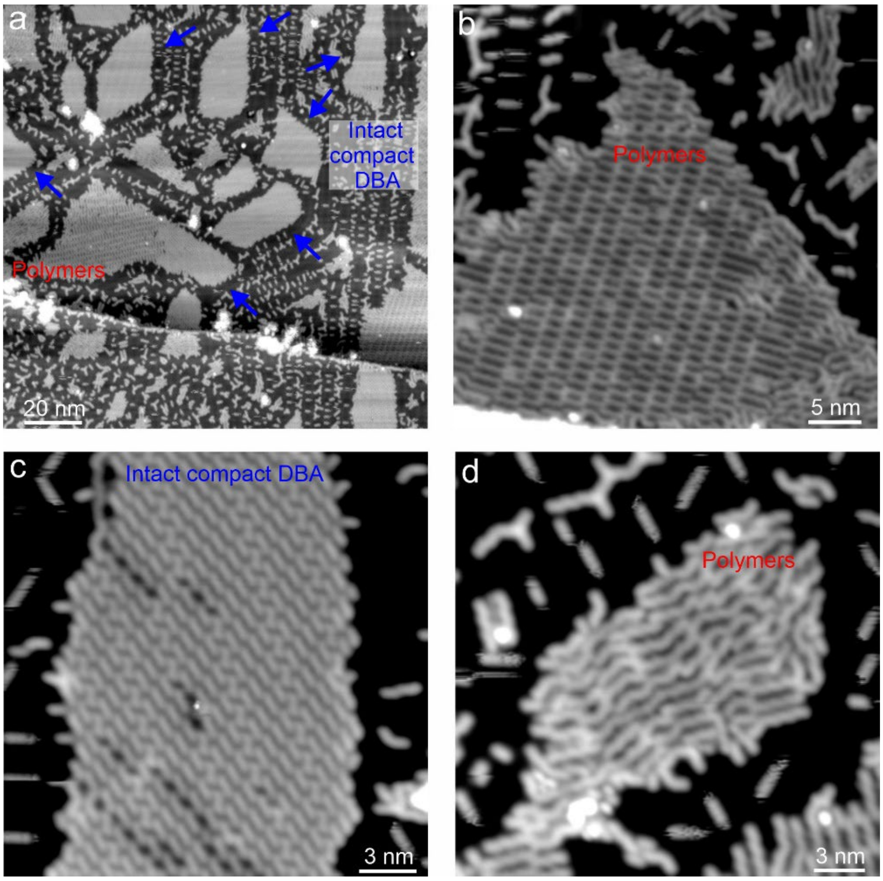

Figure S5. (a) Large STM overview of the 0.5 ML DBA/Au(111) after 3 hours of illumination at 266 nm taken at the laser spot center (see inset of Fig.S4a). (b-d) Zoom-in STM images. (b) Extended ordered islands made of polymers. (c) Compact arrangement of intact DBA molecules. (d) Disordered arrangement of polymers. Setpoints: (a: 1 V, 14 pA; b-d: 0.2 V, 100 pA).

After 3 hours of UV (266 nm) illumination extended and ordered islands comprised of polymers are found (Figs.S5a,b). Polymers can form disordered structures too (Fig.S5d). In all cases polymers are separated from each other by Br atoms rows. Compact arrangements of intact DBA molecules are also found (Fig.S5c) as a signature of the non-completed photo-debromination. Close-packed islands made of polymers and intact DBA molecules reveal a very different imaging contrast.

6. STM imaging after 7 hours of UV illumination - 0.5 ML DBA/Au(111)

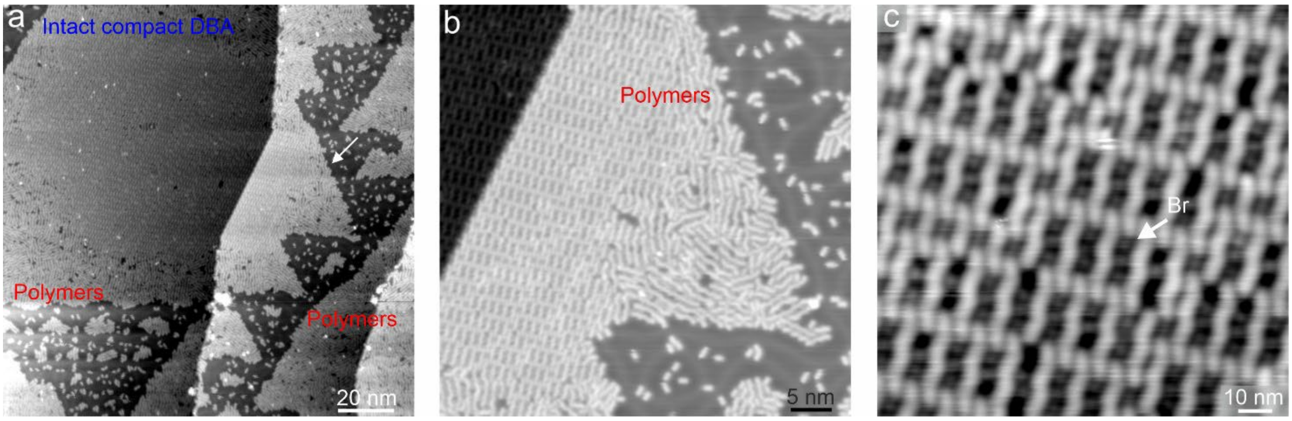

Figure S6. (a) Large STM overview of the 0.5 ML DBA/Au(111) after 7 hours of illumination at 266 nm taken at the laser spot center (see inset of Fig.S4a). (b-c) Zoom-in taken from STM image (a), see arrow. (b) Extended islands comprised of close-packed polymers. Ordered (left) and disordered (right) assembly of polymers are identified. (c) Zoom-in of the ordered close-packed assembly of dimers separated from each other by individual Br atoms. Setpoints: (a: 1 V, 11 pA; b: 0.5 V, 100 pA and c: 0.25 V, 100 pA).

Ordered and disordered close-packed structures of polymers are found (Fig.S6) after 7 hours of UV illumination. Polymers are separated from each other by Br atoms rows. Close-packed islands of intact DBA molecules are found too (not shown).

7. STM imaging after 22 hours of UV illumination - 0.5 ML DBA/Au(111)

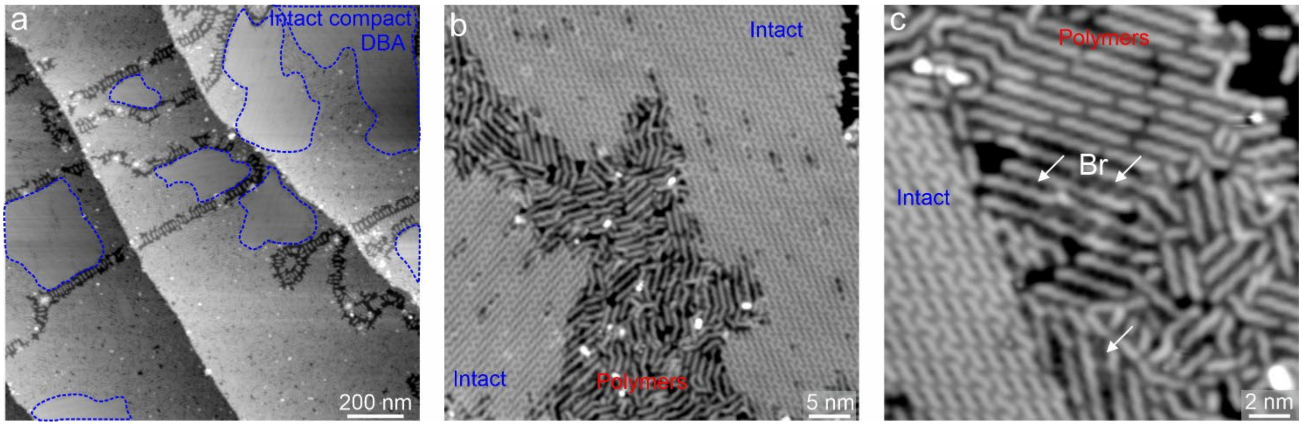

Figure S7. (a) Large STM overview of the 0.5 ML DBA/Au(111) after 22 hours of illumination at 266 nm taken at the laser spot center (see inset of Fig.S4a). Extended compact structures of intact DBA molecules are highlighted by dashed blue lines, while the rest of the islands are made of polymers. (b-c) Zoom-in STM images. (b) Close-packed polymers (in the center) surrounded by large assembly of intact DBA molecules. (c) Assembly of close-packed polymers of different length (right). Polymers are separated from each other by individual Br atoms. A cluster made of close-packed intact DBA molecules is visible at the left. Setpoints: (a: 1 V, 13 pA and b,c: 0.5 V, 80 pA).

Extended compact structures comprised of intact DBA molecules and (ordered/disordered) polymers are found (Fig.S7a). Polymers are separated from each other by Br atoms rows (see Fig.S7b,c). After 22 hours of UV (266 nm) illumination the local average molecular surface density is significantly higher compared to the value obtained before the illumination (see also Fig.4 in the main text).

8. Position-dependent STM imaging – 0.5 ML DBA/Au(111)

Before UV illumination intact DBA molecules are found arranged in extended porous networks all across the sample (Fig.S8a). After the sample was illuminated for 21 hours with UV light at RT, different sample areas across the illuminated surface area (Fig.S8g) were STM imaged at 5 K. When approaching the surface areas at the laser beam center (Fig.S8d-f), extended islands comprised either of intact DBA molecules or polymers are found. Compact structures comprised of intact DBA species are the dominant arrangements at the rim of the sample illuminated area (Fig.S8b,c).

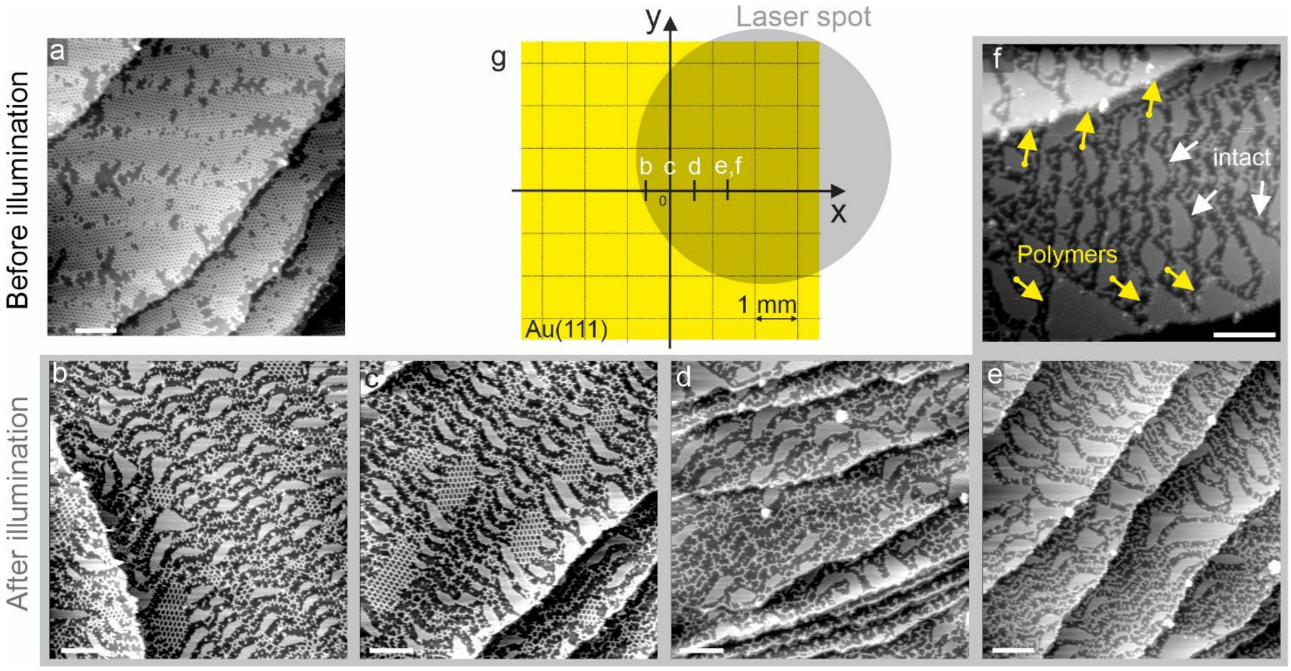

Figure S8. (a) Large STM overview of the as-prepared 0.5 ML DBA/Au(111). (b-e) Large STM overviews of the surface at different positions after 21 hours of continuous illumination at 266 nm. (f) zoom-in taken around the surface area (e). (g) Sketch indicating the positions at which the surface was STM imaged. The STM images (b-f) are accordingly labelled. The gray circle area in panel (g) shows the laser beam spot (the diameter is about 6 mm) position compared to the Au crystal (crystal size = 7 mm × 7 mm). The UV laser beam position compared to the sample has been previously identified by using a ceramic sample. Distances from the origin: (b) -0.57 mm, (c) 0 mm, (d) 0.57 mm, (e, f) 1.35 mm, y= 0 in all cases. Setpoints: (a,e: -1 V, 34 pA; b-d: -1 V, 13 pA; f : -1 V, 21 pA). The scale bar is 25 nm. STM operated at 5 K.

9. Percentage of intact DBA molecules vs UV illumination time

Intact DBA molecules are found assembled in close-packed structures by STM imaging even after long illuminations (Fig.4 and Fig.S4). Accordingly, C 1s and Br 3d core levels always show a residual covalent C–Br component. Hence, both STM and XPS measurements (conducted in different experimental setups) reveal an uncompleted light-induced debromination of the adsorbed DBA species even after several hours of UV illumination.

The intensity of the molecule-bound Br–C component (Br 3d component at high BE) is extracted from the fits of the individual Br 3d spectra. These spectra were fitted using Voigt doublet lineshapes[1] (see also pag.S4). The area of the covalent Br–C component is normalized to the total area of the Br 3d core level and then plotted as a function of the UV illumination time for two different DBA molecular coverages, 0.5 and 1.0 ML (Fig.S9). The amount of intact DBA species decreases with the illumination time, owing to the ongoing debromination process. The experimental curves are fitted by an exponential decay function: $y(t)= A + B*\exp(-t/\tau)$ - see fittings curves (dashed lines) in Fig.S9 where A corresponds to the asymptotic value.

A non-zero asymptotic value A is found for both molecular coverages. The two curves do not converge to a similar asymptotic value. The asymptotic amount of intact DBA species is about 22 % at a coverage of 1.0 ML and it is significantly reduced down to 6 % at 0.5 ML coverage. These spectroscopic findings suggest that the DBA debromination rate is influenced by the initial DBA molecular coverage. The debromination process is never brought to completion. This can be tentatively ascribed to steric constraints that play an even more substantial role at high coverages. Moreover, an equilibrium between the debromination and re-bromination processes of the adsorbed species can´t be ruled out[10].

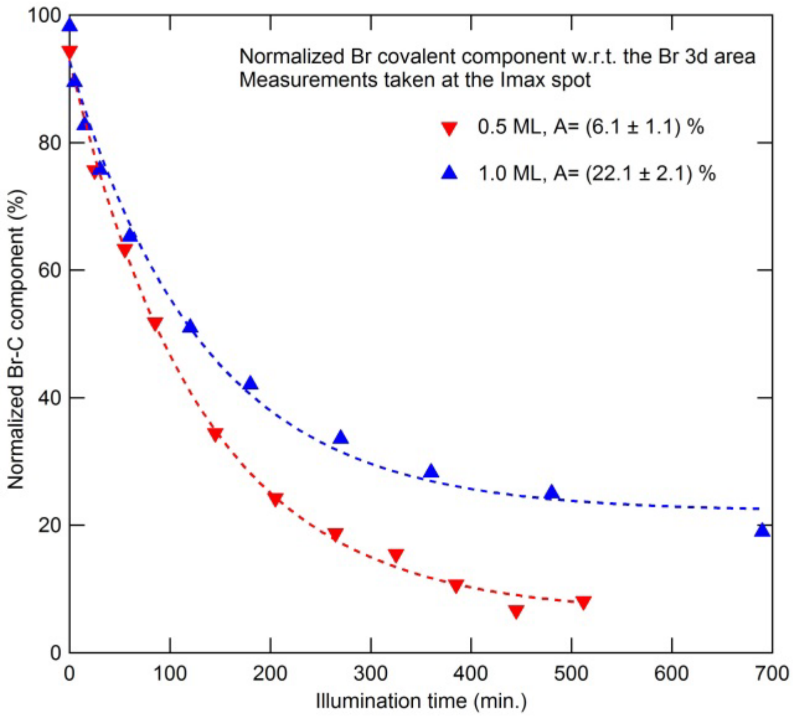

Figure S9. The normalized area of the covalent component Br–C of the Br 3d core level is plotted as a function of the UV illumination time for two different DBA molecular coverages on Au(111) (0.5 ML (red) and 1.0 ML (blue)). The Br–C component areas are normalized to the entire Br 3d peak area. The curves were fitted with an exponential decay function ($y(t)= A + B*\exp(-t/\tau)$) to estimate the asymptotic value A, i.e., the asymptotic amount of intact molecules. All Br 3d spectra were acquired at the center of the I_{max} spot.

10. Position-dependent STM imaging – 0.1 ML DBA/Au(111)

Position-dependent STM measurements have been used to probe the effect of prolongated UV illumination in the case of a low surface molecular coverage of 0.1 ML, which is a significantly lower coverage than in the main text (0.5 ML). Small clusters comprised of few DBA molecules are identified before the illumination (Fig.S10a).

The surface shown in Fig.S10a has been illuminated for 12 hours with UV (266 nm) laser light at RT. Small 2D islands comprised either of intact molecules or polymers are found (Fig.S10b-c). The light-driven clustering effect is evident when approaching the surface areas close to the laser spot center (Fig.S10d). Accordingly, the local increase of the molecular density is observed even for this low coverage.

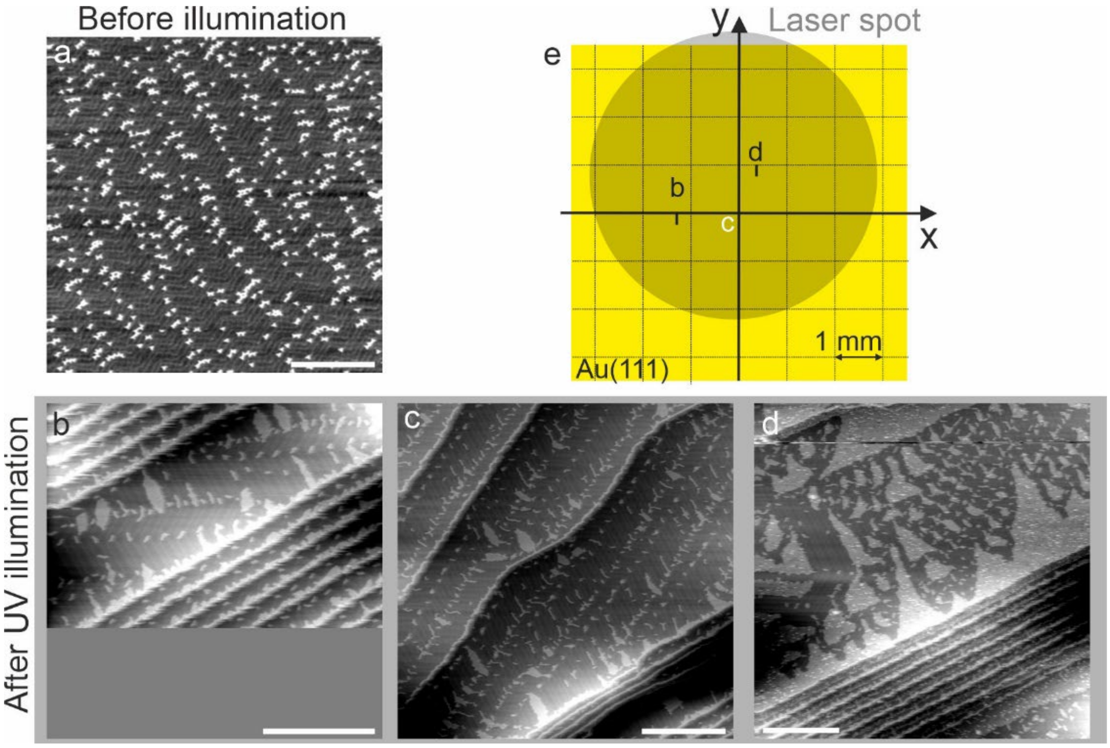

Figure S10. (a) 0.1 ML DBA on Au(111) – before the illumination (about 10% of the crystal surface is covered by molecules). (b-d) Position-dependent STM measurements of the same sample after 12 hours of continuous illumination with UV (266 nm) laser light with the sample kept at RT. (e) Sketch indicating the positions at which the surface was STM imaged after the illumination. The STM images are labelled accordingly. The gray circle in panel (e) shows the incident laser beam spot (its diameter is about 6 mm) position compared to the Au crystal (crystal size: 7 mm × 7 mm). The laser beam has been previously aligned by using a ceramic sample. Setpoints: (a,c: -1 V, 10 pA; b: -0.98 V, 20 pA; d: 1 V, 10 pA). The scale bar is 50 nm. STM operated at 5 K.

11. C 1s and Br 3d core levels vs illumination time – 1.0 ML DBA/Au(111)

C 1s and Br 3d core level spectra have been acquired at the center of the I_{max} spot in the case of 1.0 ML of DBA/Au(111) after 690 minutes of UV (266 nm) illumination (Fig.S11a,b). The intensity of the C–Br component at high BE (Fig.S11a) is progressively reduced because of the ongoing photo-driven debromination. The Br 3d reveals a single spin-orbit doublet assigned to a single chemical bromine species before the illumination (black curve in Fig.S11b) – DBA molecules are mainly intact after adsorption on Au(111) at RT. Upon irradiation a second doublet is emerging at lower binding energy (BE). The latter is ascribed to chemisorbed Br atoms[1,5,6] (see discussion in S4) and grows at the expense of the covalent component at high BE (Fig.S11d). The area of the C 1s and Br 3d core levels (Fig.S11c) were deduced by spectra fitting (see discussions about Fig.S2).

The C 1s core level area increases just by a factor of 1.20 because of the UV illumination (Fig.S11c). An increase by about a factor of 2.0 is observed in the case of 0.5 ML DBA/Au(111) (Fig.S2). The light-induced increase of the molecular surface density appears to be less prominent at higher molecular surface coverages (see Fig.S2). The debromination process also in this case does not reach completion during the experimental time though the significant longer illumination time. As observed in the case of 0.5 ML DBA/Au(111) the Br signal slowly decreases after reached a maximum intensity (Fig.S11c) (see discussions in S3-S4).

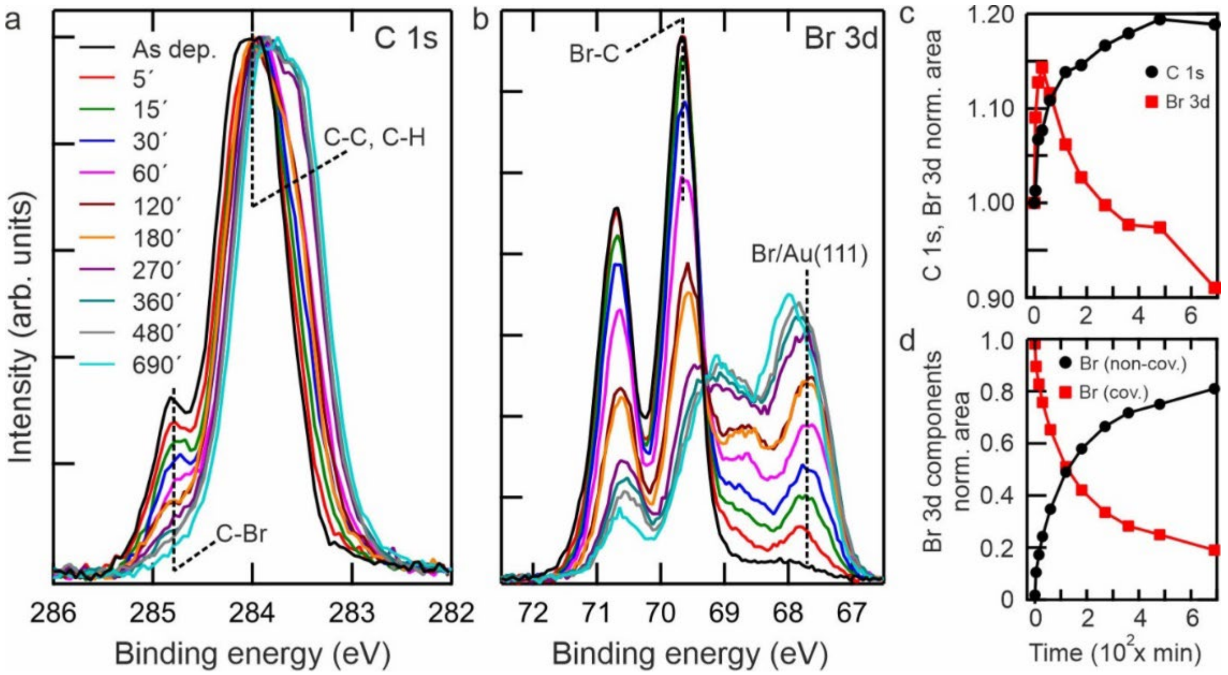

Figure S11. C 1s (a) and Br 3d (b) core level spectra measured on 1.0 ML DBA/Au(111) as a function of the UV illumination time (in minutes). All spectra are acquired at RT at the surface area corresponding to the center of the I_{max} spot. The core level components and their energy positions are indicated. (c) Normalized peak areas of C 1s and Br 3d as a function of the illumination time. All spectra areas are normalized to the peak area value before the irradiation (time zero). (d) Normalized area of the covalent and non-covalent Br 3d components as a function of the UV illumination time. They are normalized to the entire area of the Br 3d peak.

12. STM imaging of DBA/Au(111) at different temperatures

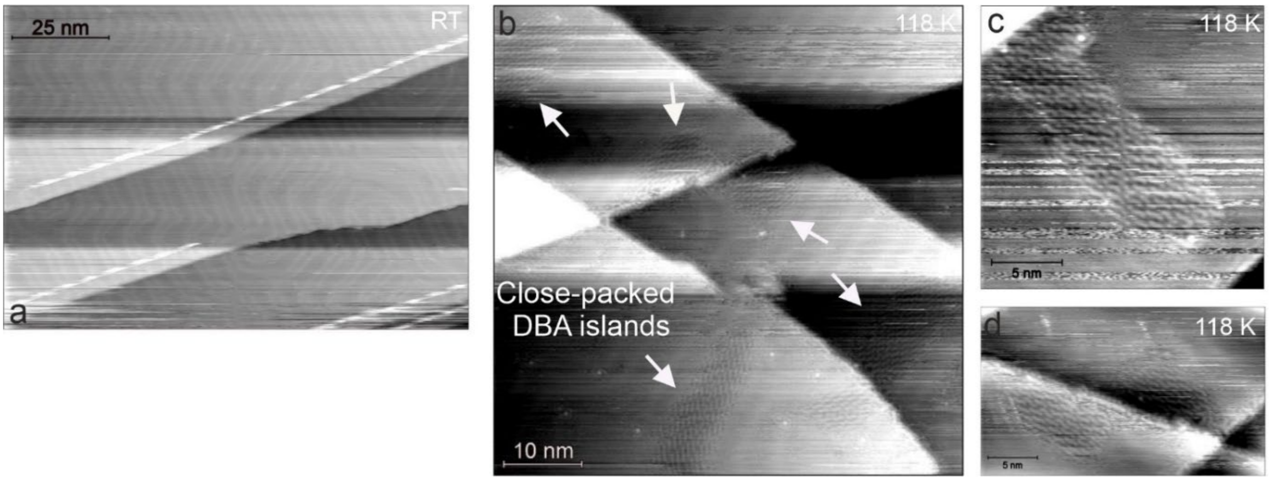

Figure S12. (a) DBA molecules on Au(111) imaged at RT. Molecules were first deposited on Au(111) kept at RT and then imaged at the same temperature. No evidence of molecular clusters (a) because molecules diffuse during scanning owing to their high mobility on Au(111) at RT. (b-d) DBA molecules on Au(111) imaged at 118 K. DBA molecules diffuse significantly less across the surface at 118 K: Compact DBA clusters and DBA molecules adsorbed on the Au steps are stably imaged. Setpoints: (a: 0.3 V, 200 pA and b-d: 0.1 V, 50 pA).

STM imaging at RT does not reveal any evidence of molecular clusters (Fig.S12a) owing to the high mobility of the molecules on Au(111). Afterward the sample was cooled down to 118 K where DBA molecules diffuse significantly less. Accordingly, parts of DBA clusters and DBA molecules adsorbed on the Au steps are stably imaged (see S12b-d). The molecules are assembled into close-packed structures (Fig.S12b-d). DBA molecules are arranged into porous networks when deposited on Au(111) kept at room temperature and quickly (30 minutes) cooled down to low temperature (5 K, 7.5 K) after being transferred in the STM (see Fig.4a in the main text). The close-packed structures observed at 118 K are likely due to the higher thermal energy of the molecules compared to the previous mentioned preparations at lower temperatures.

13. Effects due to exposure to the synchrotron beam

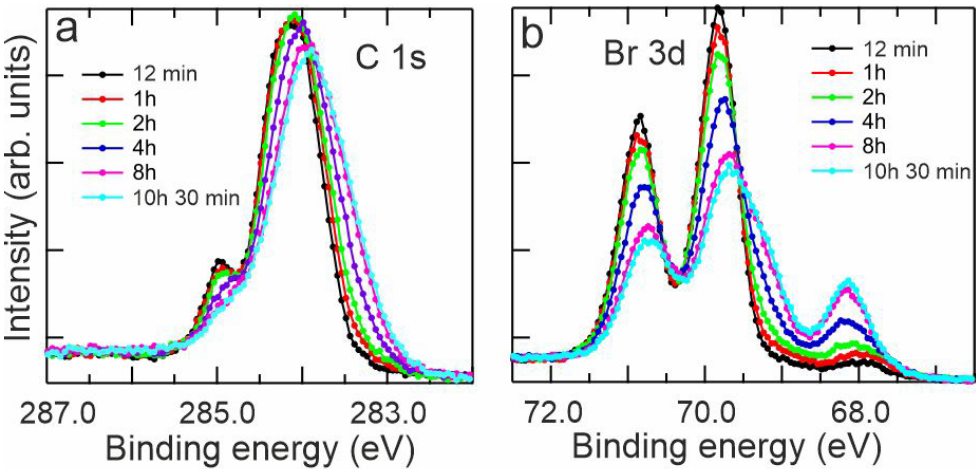

Figure S13. (a) C 1s and (b) Br 3d probed as a function of the exposure to the synchrotron beam. C 1s and Br 3d have been acquired in continuous mode for 10h30min. Few selected spectra are shown in the panels to highlight the X-ray beam-induced modifications of the core levels.

It is known that intense synchrotron radiation light can induce chemical modifications of the adsorbed species. To explore the X-ray beam-induced effects, C 1s and Br 3d XPS spectra ($h\nu = 380$ eV) were acquired in continuous mode for 10h30min (Fig. S13) where X-ray beam-induced modifications of the spectra are visible. In order to minimize such exposure effects, the spectra acquisition time has been chosen so that the core levels are not modified, but statistics still guarantee a suitable noise level. The core level acquisition time typically ranged from few minutes up to a maximum 15 minutes, which is all shorter than the time needed to induce significant modifications (about 1 hour, see Fig. S13). This allows to reasonably minimize the influence of the synchrotron beam.

References

1. Nacci, C. *et al.* Thermal- vs Light-Induced On-Surface Polymerization. *J. Phys. Chem. C* **125**, 22554–22561 (2021).

2. Simonov, K. A. *et al.* Effect of substrate chemistry on the bottom-up fabrication of graphene nanoribbons: Combined core-level spectroscopy and STM study. *J. Phys. Chem. C* **118**, 12532–12540 (2014).

3. Basagni, A. *et al.* Molecules − Oligomers − Nanowires − Graphene Nanoribbons: A Bottom-Up Stepwise On-Surface Covalent Synthesis Preserving Long-Range Order. *J. Am. Chem. Soc.* **137**, 1802–1808 (2015).

4. Abyazisani, M., MacLeod, J. M. & Lipton-Duffin, J. Cleaning up after the Party: Removing the Byproducts of On-Surface Ullmann Coupling. *ACS Nano* **13**, 9270–9278 (2019).

5. Doyle, C. M. *et al.* Surface Mediated Synthesis of 2D Covalent Organic Networks: 1,3,5-Tris(4-bromophenyl)benzene on Au(111). *Phys. Status Solidi Basic Res.* **256**, 1–11 (2019).

6. Smykalla, L., Shukrynau, P., Korb, M., Lang, H. & Hietschold, M. Surface-confined 2D polymerization of a brominated copper-tetraphenylporphyrin on Au(111). *Nanoscale* **7**,

4234–4241 (2015).

7. Salvo, C. R., Keagy, J. & Yarmoff, J. A. Adsorption of Br2 onto Small Au Nanoclusters. *J. Phys. Chem. C* **122**, 24732–24739 (2018).

8. Moreno, C. *et al.* Critical Role of Phenyl Substitution and Catalytic Substrate in the Surface-Assisted Polymerization of Dibromobianthracene Derivatives. *Chem. Mat.* **31**, 331–341 (2019).

9. Smerieri, M. *et al.* Synthesis of graphene nanoribbons with a defined mixed edge-site sequence by surface assisted polymerization of (1,6)-dibromopyrene on Ag(110). *Nanoscale* **8**, 17843–17853 (2016).

10. Fritton, M. *et al.* The Role of Kinetics versus Thermodynamics in Surface-Assisted Ullmann Coupling on Gold and Silver Surfaces. *J. Am. Chem. Soc.* **141**, 4824–4832 (2019).
